# Construction of gene modification system with highly efficient and markerless for *Monascus ruber* M7

**DOI:** 10.3389/fmicb.2022.952323

**Published:** 2022-08-01

**Authors:** Na Xu, Li Li, Fusheng Chen

**Affiliations:** ^1^Hubei International Scientific and Technological Cooperation Base of Traditional Fermented Foods, Huazhong Agricultural University, Wuhan, China; ^2^College of Food Science and Technology, Huazhong Agricultural University, Wuhan, China; ^3^Hubei Key Laboratory of Quality Control of Characteristic Fruits and Vegetables, Hubei Engineering University, Xiaogan, China; ^4^College of Life Science and Technology, Hubei Engineering University, Xiaogan, China

**Keywords:** *Monascus ruber* M7, genetic modification system, resistance selection marker, *mrlig4*, *mrpyrG*

## Abstract

*Monascus* spp. are traditional medicinal and edible filamentous fungi in China, and can produce various secondary metabolites, such as *Monascus* pigments (MPs) and citrinin (CIT). Genetic modification methods, such as gene knock-out, complementation, and overexpression, have been used extensively to investigate the function of related genes in *Monascus* spp.. However, the resistance selection genes that can have been used for genetic modification in *Monascus* spp. are limited, and the gene replacement frequency (GRF) is usually <5%. Therefore, we are committed to construct a highly efficient gene editing system without resistance selection marker gene. In this study, using *M. ruber* M7 as the starting strain, we successfully constructed a so-called markerlessly and highly genetic modification system including the mutants Δ*mrpyrG*Δ*mrlig4* and Δ*mrpyrG*Δ*mrlig4*::*mrpyrG*, in which we used the endogenous gene *mrpyrG* from *M. ruber* M7 instead of the resistance marker gene as the screening marker, and simultaneously deleted *mrlig4* related to non-homologous end joining in *M. ruber* M7. Then, the morphology, the growth rate, the production of MPs and CIT of the mutants were analyzed. And the results show that the mutant strains have normal mycelia, cleistothecia and conidia on PDA+Uridine(U) plate, the biomass of each mutant is also no different from *M. ruber* M7. However, the U addition also has a certain effect on the orange and red pigments yield of *M. ruber* M7, which needs our further study. Finally, we applied the system to delete multiple genes from *M. ruber* M7 separately or continuously without any resistance marker gene, and found that the average GRF of Δ*mrpyrG*Δ*mrlig4* was about 18 times of that of *M. ruber* M7. The markerlessly and highly genetic modification system constructed in current study not only will be used for multi-gene simultaneous modification in *Monascus* spp., and also lays a foundation for investigating the effects of multi-genes modification on *Monascus* spp..

## Introduction

*Monascus* spp., a group of traditional medicine and edible filamentous fungi in China, can produce abundant benefit secondary metabolites (SMs) such as *Monascus* pigments (MPs), γ-aminobutyric acid, monacolin K, and ergosterol (Endo, 1979; Feng et al., 2012; Patakova, 2013; Wu et al., 2013). But some *Monascus* strains may also yield a kind of mycotoxin, citrinin (CIT) (Blanc et al., 1995; Lin et al., 2008). So, there are a lot of studies on how to improve benefit SMs amount and to decrease and even eliminate CIT content in *Monascus-*related products (de Carvalho et al., 2006; Hajjaj et al., 2012; Feng et al., 2014). Among them, the genetic modification method such as gene knock-out, complementation, and overexpression is considered as one of the most significant approaches to control benefit SMs and CIT production (Liu et al., 2014, 2016, 2021; Zhang et al., 2019).

However, as we know, up to now, there are limited antibiotic selection marker genes available for the genetic modification of *Monascus* spp., such as the genes of hygromycin (*hph*), neomycin (*neo*) (Li, 2011), pyrithiamine (*pyr*) (Cui and Li, 2012), and aureobasidin A (*aba*) (Shimizu et al., 2006). Therefore, it is very difficult to simultaneously modify multiple genes in the same *Monascus* strain. Moreover, the antibiotic resistance marker genes remaining in the *Monascus* mutants may affect their growth and metabolism, and when the mutants are used in the production of foods and food additives, there also exist potential food safety hazards (Tuteja et al., 2012; Yang et al., 2015). So, the development of the genetic modification method without antibiotic screening marker gene residues is requisite for *Monascus* gene modification.

The uridine (U) auxotroph has been exploited widely in the genetic transformation system for many filamentous fungi (Wang et al., 2010; Arentshorst et al., 2015; Huang et al., 2016; Nguyen et al., 2016; Zhang et al., 2020). In fungi, orotidine 5′-phosphate decarboxylase (OMP decarboxylase) encoded by *pyrG* gene is a key enzyme involved in the pyrimidine biosynthesis, which can catalyze the decarboxylation of OMP to form uridine monophosphate (UMP) (Caroline and Davis, 1969; Garavaglia et al., 2012). OMP decarboxylase can also transform the pyrimidine analog 5-fluoroorotic acid (5-FOA) to the toxic compound to fungi, 5′-fluoro-UMP, to kill the fungal cells with *pyrG* (Ying et al., 2013; Zhang et al., 2020). Therefore, U combined with 5-FOA can be used to screen a markerless knockout strain, Δ*pyrG*, which does not contain the foreign gene including any antibiotic selection marker gene. The strains containing *pyrG* can synthesize U by themselves and can grow on the media without U, so they are called as U prototrophic or U independent strains, while the strains without *pyrG* such as Δ*pyrG* cannot synthesize U by themselves and also cannot grow on the media without U, so they are known as U auxotrophic or U-dependent strains. Therefore, taking Δ*pyrG* as the starting strain, replacing the target gene with *pyrG*, and combining U with 5-FOA, the markerless modifier of the target gene can be achieved. Wang et al. (2010) obtained a *pyrG* gene point mutant strain of *M. aurantiacus* by ultraviolet mutagenesis, and successfully transferred *pyrG* back to the *pyrG* mutant strain. However, there is no report about their subsequent application research of this system.

Several studies have showed that the gene homologous recombination efficiency (GRF) of fungal gene modification is relatively low due to the existence of the non-homologous end-joining (NHEJ) pathway in fungal cells (Ishibashi et al., 2006; Shrivastav et al., 2008; Liu et al., 2018; Pannunzio et al., 2018). And usually, the GRF of *Monascus* genetic modification is <5% (Li and Chen, 2020). He et al. (2013, 2014) knocked out the relative genes with NHEJ pathway, including the genes of DNA-dependent protein kinase catalytic subunits of Ku70 and Ku80, and ligase 4 (Symington and Gautier, 2011), leading that GRF of *Monascus* was increased by 2–4 times. However, the obtained high-efficiency strains cannot continue to be used for multi-gene modification of *M. ruber* M7 due to the limitation of resistance screening genes.

In current research, we have developed a marker recycling and highly genetic modification system, including the mutants Δ*mrpyrG*Δ*mrlig4*::*mrpyrG* (Δ*pyrG*+*lig4::pyrG*) and Δ*mrpyrG*Δ*mrlig4* (Δ*pyrG*+*lig4*) for *M. ruber* M7, in which we used the endogenous gene *mrpyrG* (the homologous gene of OMP decarboxylase gene) in *M. ruber* M7 instead of the resistance marker gene as the screening marker, and simultaneously deleted *mrlig4* related to the NHEJ. Then, the morphologies, growth rates, the production of MPs and CIT of the mutants were determined. Finally, we applied the system to delete multiple genes including *mrpigG, mrpigH*, and *mrpigI* relative to MPs biosynthesis in *M. ruber* M7 (Chen et al., 2017) separately or continuously without any resistance marker gene, and found that the average GRF of Δ*pyrG*+*lig4* was about 18 times of that of *M. ruber* M7, which shows that the system is suitable for multiple genes modification of *Monascus* spp..

## Materials and methods

### Fungal strains, culture media and growth conditions

*M. ruber* M7 [CCAM 070120, Culture Collection of State Key Laboratory of Agricultural Microbiology, which is part of China Center for Type Culture Collection (CCTCC), Wuhan, China] (Chen and Hu, 2005), the model microorganism in our lab, was used as a DNA donor and for transformation (Shao et al., 2009). All the strains used in this study are described in Table 1. All the strains are maintained on PDA slants with/without 10 mmol/ml uridine and 0.75 mg/ml 5-FOA at 28°C (Thai et al., 2021).

**Table 1:** *Monascus ruber* strains used and constructed in this study.

**Strains**	**Parents**	**Sources**
***M. ruber* M7**	**–**	**Laboratory preservation**
**Δ*mrpyrG***	***M. ruber* M7**	**This study**
**Δ*mrlig4*Δ*mrpyrG* (Δ*pyrG*+*lig4*)::*mrpyrG***	**Δ*mrpyrG***	**This study**
**Δ*pyrG*+*lig4***	**Δ*pyrG*+*lig4*::*pyrG***	**This study**
**Δ*pyrG*+*li*g4+*pigG***	**Δ*pyrG*+*lig4***	**This study**
**Δ*pyrG*+*li*g4+*pigI***	**Δ*pyrG*+*lig4***	**This study**
**Δ*pyrG*+*lig4*+*pigG*+*pigH***	**Δ*pyrG*+*li*g4+*pigG***	**This study**
**Δ*pyrG*+ *lig4*+*pigG*+*pigH*+*pigI***	**Δ*pyrG*+*lig4*+*pigG*+*pigH***	**This study**

### Cloning and analysis of the *pyrG* gene

Amino acid sequences encoded by *mrpyrG* were predicted using SoftBerry's FGENESH program (http://www.softberry.com), and the *mrpyrG* functional regions were analyzed using the Pfam 33.1 program (http://pfam.xfam.org/). Homology of the deduced amino acid sequence was analyzed using the BlastP program on the NCBI website (http://blast.ncbi.nlm.nih.gov/Blast.cgi).

### Deletion of the *mrpyrG* and *mrlig4* genes

**Figure 1:**
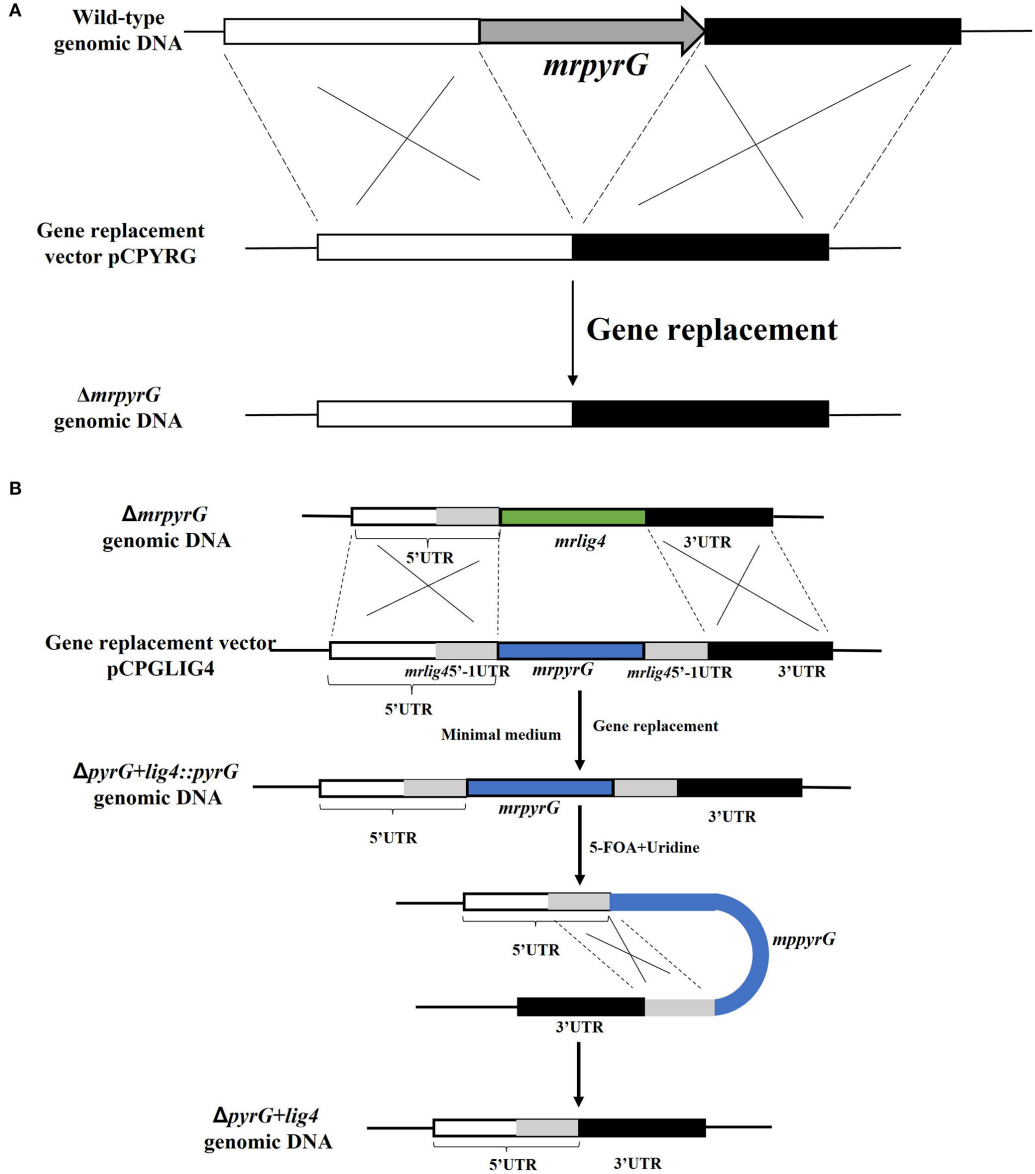
Deletion of *mrpyrG* and *mrlig4* in *M. ruber* M7. **(A)** Strategy to construct *mrpyrG* markerless deletion strain. **(B)** Strategy to construct *mrlig4* markerless deletion strain.

To construct a markerlessly and highly efficient genetic modification system, the genes of *mrpyrG* and *mrlig4* were deleted according to the homologous recombination strategy as described previously (Liu et al., 2014). Genomic DNA of *M. ruber* M7 was extracted according to previous description (Shao et al., 2009) for amplification of the entire *mrpyrG* and *mrlig4* gene sequences and their 5′- and 3′-flanking regions. Using these amplified DNA sequences, the *mrpyrG* deletion cassette was constructed by double-joint PCR (Yu et al., 2004), and the *mrlig4* cassette was constructed using the Seamless Cloning and assembly kit (Li et al., 2021). Then, these two cassettes were digested separately by *Kpn* I/*Xba* I, *Hind* III/*Kpn* I, and then ligated with pCAMBIA3300 vector digested with the same restricted enzymes to form recombinant vectors respectively, which were transformed into *Agrobacterium tumefaciens* EHA105 cells that were used to introduce the constructed cassettes region into the hosts (Shao et al., 2009). The construction procedure is showed in Figure 1 (Li et al., 2021). The relative primer pairs are shown in Table 2.

**Table 2:**
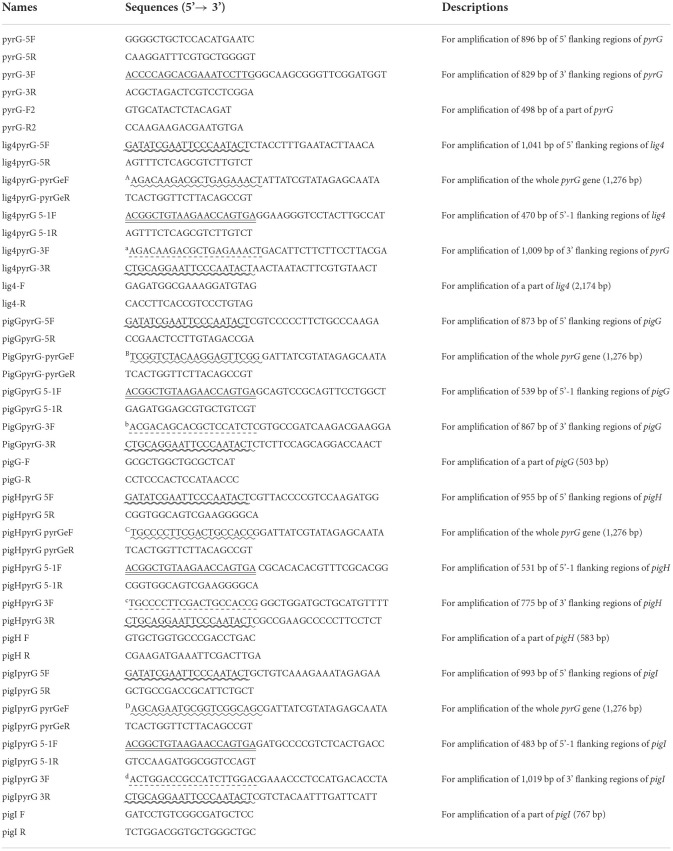
Primers used in this study.

*Labeled with double wavy line letters are nucleotide sequences of pBLUE-T; Labeled with single underline letters are nucleotide sequences of 5'UTR of mrpyrG; Labeled with double underline letters are nucleotide sequences of mrpyrG; ^A, B, C, D^Labeled with wavy line letters are nucleotide sequences of 5′ flanking regions of mrlig4, mrpigG, mrpigH, and pigI, respectively; ^a, b, c, d^Labeled with dotted lines letters are nucleotide sequences of 5′-1 flanking regions of mrlig4, mrpigG, mrpigH, and mrpigI, respectively*.

### Excision of the *mrpyrG* marker by using 5-FOA

Since the U auxotrophs were resistant to 5-FOA in *M. ruber* M7, positive selection for Δ*mrpyrG* strains was carried out using 5-FOA. It was expected that the *pyrG* inserted at the *lig4* locus would be excised out by homologous recombination with the direct repeats, in which the flanking regions of the *lig4* were directly connected without leaving any ectopic/foreign DNA fragments (Figure 1B). Conidia of the Δ*pyrG*+*lig4*::*pyrG* strains(10^5^ cfu/ml) were spread onto the agar medium containing 5-FOA and U after 5–8 day cultivation and the resulting 5-FOA resistant strains exhibited U auxotrophy.

### Analysis of phenotypic characterization and biomass

*M. ruber* M7, Δ*mrpyrG*, Δ*pyrG*+*lig4*::*pyrG*, Δ*pyrG*+*lig4*, were, respectively, inoculated on PDA, PDA+U, PDA+U+5-FOA plates for 5 days at 28°C to observe the colonial and microscopic morphologies (Huang et al., 2016).

Biomass was determined according to the published paper (Lai et al., 2011) with minor modification. One milliliter freshly harvested spore (10^5^ cfu/ml) of each strain was inoculated on PDA and PDA+U plates covered with cellophane membranes, and incubated at 28°C for 11 days, the samples were taken every 2 days from the 3rd day to the 11th day of culture. Then, these samples were vacuum freeze-dried and weighed.

### Detection of citrinin and *Monascus* pigments

In total, 1 ml freshly harvested spore (10^5^ cfu/ml) of aforementioned strains was inoculated on PDA and PDA+U plates covered with cellophane membranes, and incubated at 28°C for 11 days, respectively, to detect the intracellular MPs and extracellular CIT.40 mg freeze-dried media powder was extracted by 1 mL 80% (v/v) methanol, and subjected to 30 min ultrasonication treatment to detect the citrinin content by UPLC (Waters, America) with previous method (Liu et al., 2019), and injection volume was 2 μL. And 20 mg freeze-dried mycelia was extracted by 1 mL 80%(v/v) methanol, and subjected to 30 min ultrasonication treatment. Dilute the methanol extract to an appropriate amount, use 80% (v/v) methanol as the control (CK), and measure the absorbance at 380 nm, 470 nm, and 520 nm by the ultraviolet-visible spectrophotometry. The absorbance value multiplied by the dilution factor is the color value of yellow, orange, and red MPs, respectively. The final contents of Monascus pigments and citrinin were expressed as U/mg and μg/mg, respectively.

## Results

### Construction of genetic modification system with markerless and highly efficient system

#### Sequence analysis of *mrpyrG* in *M. ruber* M7

Sequence prediction of *mrpyrG* by SoftBerry's FGENESH program has revealed that the putative *mrpyrG* gene consists only of an 828 bp open reading frame (ORF) which consists of 2 exon and encodes 275 amino acids. A database search with NCBI-Blastp has demonstrated that the deduced 275-amino acid sequence encoded by *mryrG* shares 100% similarity with the amino orotidine-5′-phosphate decarboxylase of *M. aurantiacus* (GenBank: ADE43948.1), 81.39% similarity with PyrG of *Penicillium chrysogenum* (GenBank: XP-002 558877.1). Besides, prediction of Pfam has indicated that *MrpyrG* belongs to the DRE–TIM metallolyase superfamily.

#### Verification of the Δ*mrpyrG*, Δ*pyrG*+*lig4::pyrG*, Δ*pyrG*+*lig4* strains

**Figure 2:**
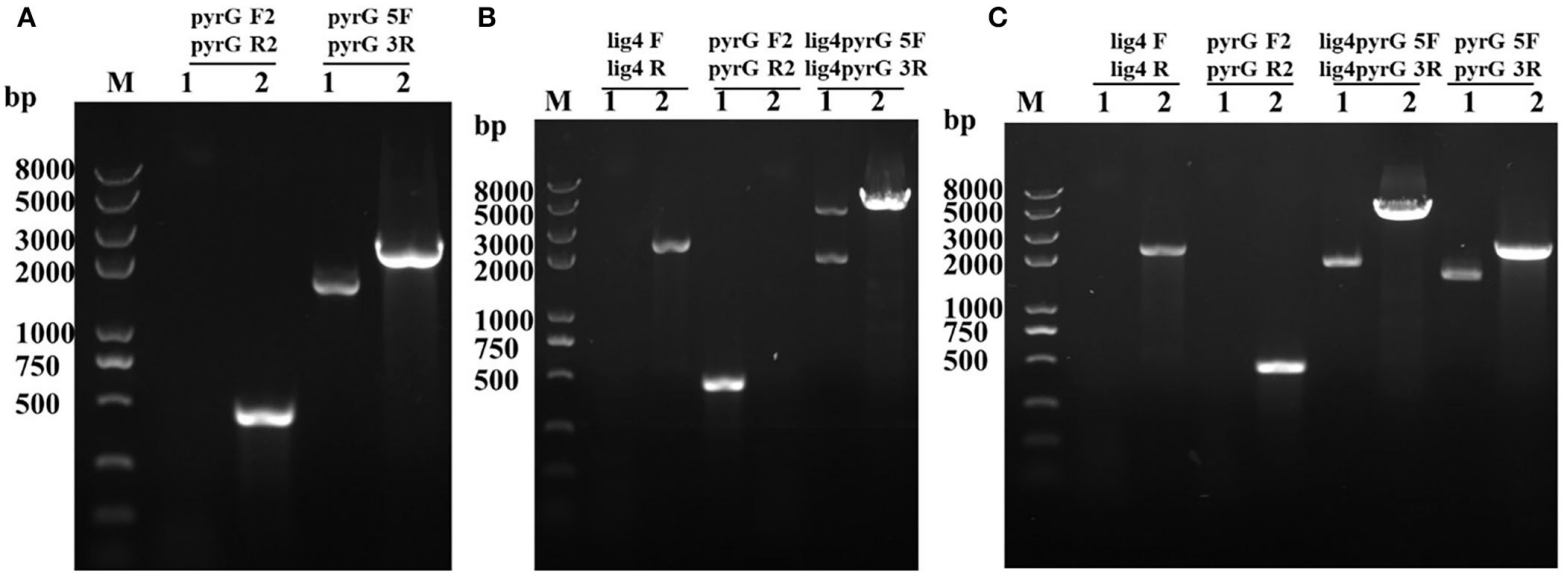
PCR analysis of Δ*mrpyrG*, Δ*pyrG*+*lig4*::*pyrG*, and Δ*pyrG*+*lig4*. **(A)** Confirmation of *mrpyrG* deletion. Lane 1: Δ*mrpyrG*; Lane 2: M7; M: Trans 2K plus II marker; **(B)** Confirmation of *mrlig4* markerless deletion in Δ*mrpyrG* strain. Lane 1: Δ*pyrG*+*lig4::pyrG*; Lane 2: Δ*mrpyrG*; **(C)** Confirmation of *mrpyrG* homologous recombination events in Δ*pyrG*+*lig4*::*pyrG* strain. Lane 1: Δ*pyrG*+*lig4*; Lane 2: M7; M: Trans 2K plus II marker.

Through genetic transformation mediated by *Agrobacterium tumefaciens*, 2 putative *mrpyrG* mutants (Δ*mrpyrG*), 2 putative mutants (Δ*pyrG*+*lig4*::*pyrG*), and 1 Δ*pyrG*+*lig4* mutant were obtained, respectively. The PCR verification results of these mutants are shown in Figure 2.

The results from Figure 2A reveal that no DNA band was amplified when the genome of the putative Δ*mrpyrG* strain was used as template with the primer pair pyrG-F2/pyrG-R2 (Table 2). Meanwhile, amplicons of *M. ruber* M7 (1.73 kb) and Δ*mrpyrG* (2.3 kb) different in sizes were observed when primers pyrG5F/pyrG3R (Table 2) were used. The results from Figure 2B show that no DNA band was amplified when the genome of the putative Δ*pyrG*+*lig4*::*pyrG* strain was used as template with the primer pair lig4F/lig4R (Table 2), while a 2.2 kb product appeared using the genome of the Δ*mrpyrG* strain. Meanwhile, amplicons of Δ*pyrG*+*lig4*::*pyrG* (3.8 kb) and Δ*mrpyrG* (4.37 kb) different in sizes were observed when primers lig4pyrG5F/lig4pyrG5R (Table 2) were used. The 2.1 kb band in Lane 1 of Figure 2B generated by the primer lig4pyrG5F/lig4pyrG5R may be the homogenous sequence of 5′UTR and 5′-1UTR of the *mrlig4* knockout cassette. The results from Figure 2C displays that no DNA band was amplified when the genome of the putative Δ*pyrG*+*lig4* strain was used as DNA template with the primer pair lig4F/lig4R and pyrGF2/pyrGR2, while a 2.2 kb product and a 0.5 kb product appeared, respectively, using the genome of the Δ*pyrG*+*lig4*::*pyrG* strain. Meanwhile, amplicons of *M. ruber* M7 (4.37kb) and Δ*pyrG*+*lig4* (2.1 kb) different in sizes were observed when primers lig4pyrG5F/lig4pyrG3R (Table 2) were used. Besides, amplicons of *M. ruber* M7 (1.73 kb) and Δ*pyrG*+*lig4* (2.3 kb) different in sizes were observed when primers pyrG5F/pyrG3R were used. These PCR results demonstrate that all the mutants are successfully constructed.

### Characteristics of *M. ruber* M7, Δ*mrpyrG*, Δ*pyrG*+*lig4*::*pyrG* and Δ*pyrG*+*lig4*

#### Morphologies and biomasses of *M. ruber* M7, Δ*mrpyrG*, Δ*pyrG*+*lig4::pyrG* and Δ*pyrG*+*lig4*

*M. ruber* M7, Δ*mrpyrG*, Δ*pyrG*+*lig4*::*pyrG* and Δ*pyrG*+*lig4* strains were cultured for 5d at 28°C to observe colonial morphologies on PDA, PDA+U, and PDA+U+ 5-FOA plates. At the same time, these strains were cultured for 7 d at 28°C to observe microscopic morphologies on PDA and PDA+U plates.

**Figure 3:**
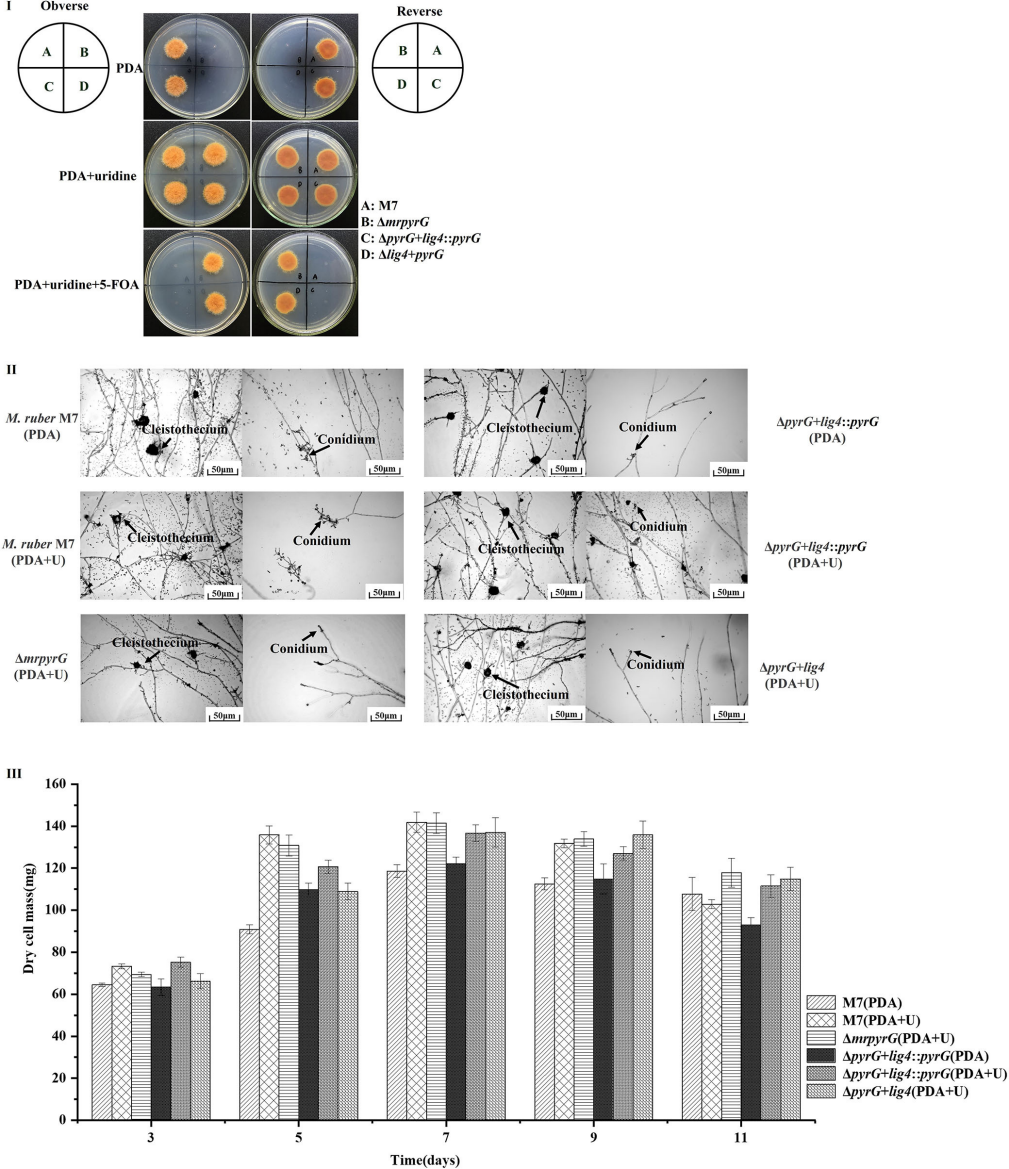
Morphologies and biomasses of *M. ruber* M7 and derivative markerless deletion strains. **(I)** Colonial morphologies on PDA, PDA+U and PDA+U+ 5-FOA plates. **(II)** Cleistothecia and conidia formation on PDA and PDA+U plates. **(III)** Biomass (dry cell weight).

It can be seen from Figure 3I that *M. ruber* M7 with *mrpyrG* can synthesize U and transform 5-FOA into the toxic compound 5-fluorouracil, so it can grow on PDA plate but not on PDA+5-FOA plate. U auxotrophic strains (Δ*mrpyrG* and Δ*pyrG*+*lig4*) cannot synthesize U and cannot transform 5-FOA into 5-fluorouracil, and cannot grow on PDA plate, but can grow on PDA+U and PDA+U+5-FOA plates. The colonial morphologies of U prototrophic strain (Δ*pyrG*+*lig4*::*pyrG*) on PDA, PDA+U, and PDA+U+5-FOA plates is consistent with *M. ruber* M7. These results once again show that the construction of each mutant is right. Meanwhile, the microscopic results in Figure 3II shows that *mrpyrG*-deficient strains (Δ*pyrG* and Δ*pyrG*+*lig4*) have normal mycelia, cleistothecia, and conidia on PDA+U plate, which are no different from *M. ruber* M7. At the same time, the U prototrophic strain (Δ*pyrG*+*lig4*::*pyrG*) also normal mycelia, cleistothecia and conidia on PDA and PDA+U plates, which is also no difference from *M. ruber* M7. Moreover, the biomass of each mutant on PDA and/or PDA+U plate is not obviously different from that of *M. ruber* M7 (Figure 3III).

#### MPs and CIT production analysis of Δ*mrpyrG*, Δ*pyrG*+*lig4::pyrG*, Δ*pyrG*+*lig4* and *M. ruber* M7

**Figure 4:**
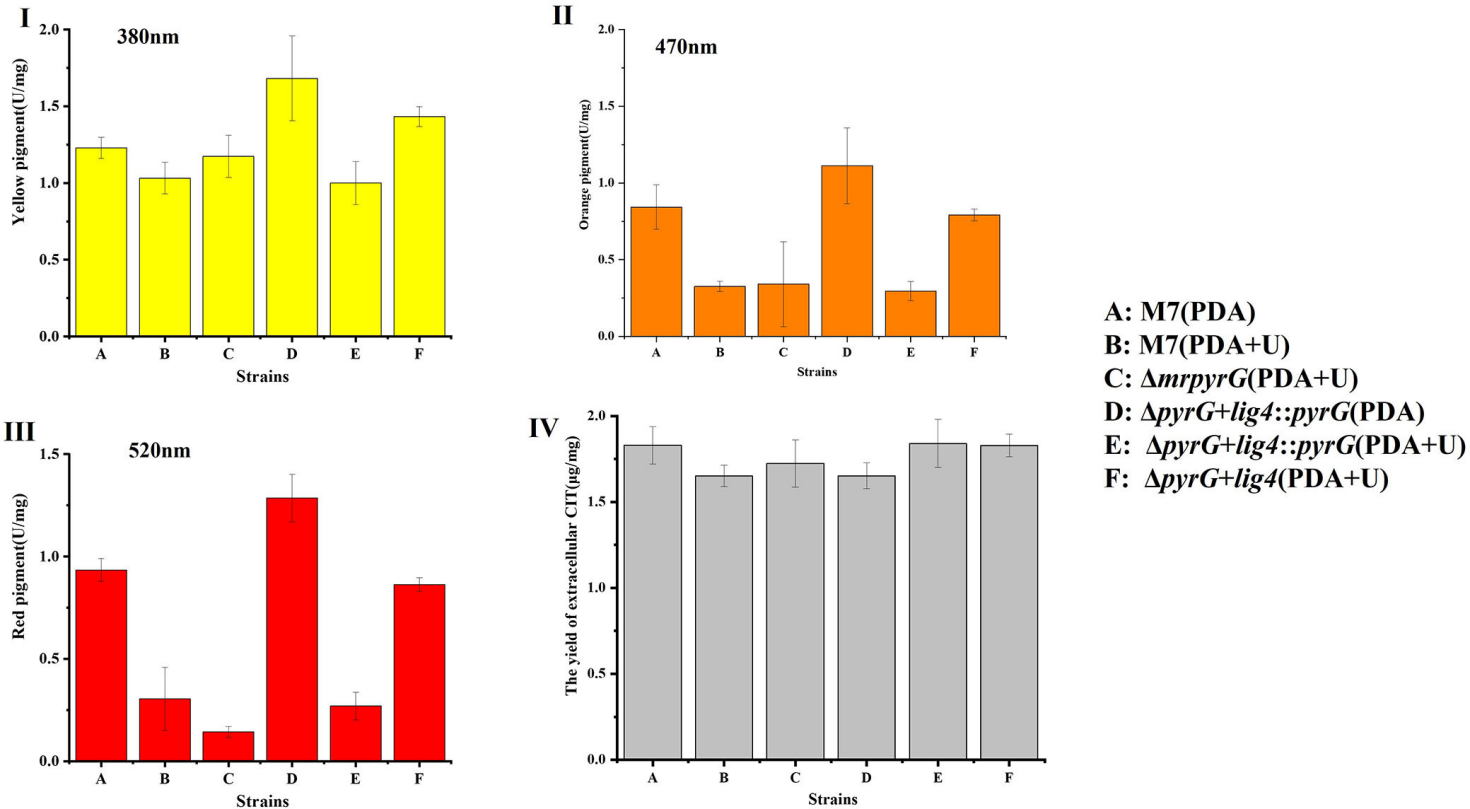
Production of intracellular MPs and extracellular CIT by *M. ruber* M7 and derivative markerless deletion strains on the PDA supplied with/without uridine. **(I)** The yield of yellow pigments; **(II)** The yield of orange pigments; **(III)** The yield of red pigments; and **(IV)** The yield of CIT.

Previous studies (Chen et al., 2017) have demonstrated that *M. ruber* M7 can produce MPs and CIT, but no MK, so the yields of MPs and CIT produced by *M. ruber* M7 and its mutants, were analyzed to uncover the effect of *mrpyrG* on them (Figure 4).

Compared with *M. ruber* M7, the yellow pigment production of all the mutant strains changed to some extent, but it showed an irregular trend (Figure 4I). And for the U auxotrophic strains Δ*mrpyrG* and Δ*pyrG*+*lig4*, the addition of U affected the production of orange and red pigments to a certain extent (Figures 4II,III). In addition, it can be found that the U addition also has a certain effect on the orange and red pigments yield of *M. ruber* M7.

With regard to CIT, the results (Figure 4IV) show that CIT produced by Δ*mrpyrG*, Δ*pyrG*+*lig4*::*pyrG* and Δ*pyrG*+*lig4* was not apparently different from those of *M. ruber* M7 on different media, which indicates that *mrpyrG* and *mrlig4* have no effect on the CIT production.

#### Application of gene markerless and highly efficient modification system

Taking *M. ruber* M7 and Δ*pyrG*+*lig4* as the starting strains to knock out *mrpigG* and *mrpigI* in the MPs gene cluster of *M. ruber* M7 (Chen et al., 2017), respectively, and calculate the number of transformants and the number of disruptants (knockouts) via PCR verification with related primers shown in Table 2 from both starting strains. And the GRFs, referred as the number of disruptants divided by the number of transformants, are shown in Table 3. Furthermore, taking Δ*pyrG*+*lig4*+*pigG* as a starting strain, *mrpigH* and *mrpigI* genes were continuously deleted, and successfully got the multi-gene mutants Δ*pyrG*+*lig4*+*pigG* +*pigH*+*pigI*, and Δ*pyrG*+*lig4*+*pigG*+*pigH*+ *pigI*::*pyrG*. As shown in Table 3, when *mrpigG* was knocked out using the Δ*pyrG*+*lig4* strain as the starting strain, the GRF reached to 46.7% (21/45), while using *M. ruber* M7 as the starting strain, the GRF of *pigG* was only 2.6% (3/115). Meanwhile, when *mrpigI* was knocked out, the GRF was 44.4% (4/9) in the Δ*pyrG*+*lig4* strain and 2.4% (2/85) in *M. ruber* M7. In general, the average GRFs for *mrpigG* and *mrpigI* in Δ*pyrG*+*lig4* was about 18 times of that of *M. ruber* M7.

**Table 3:** GRF in the wild-type (M7) and Δ*pyrG*+*lig4* strains.

**Target**	**GRF of**	**GRF of M7**
**genes**	**Δ*mrpyrG*+*lig4***	**(Disruptants** ^a^
	**(Disruptants** ^a^	**/Transformants** ^b^ **)**
	**/Transformants** ^b^ **)**	
*mrpigG*	46.7% (21/45)	2.6% (3/115)
*mrpigI*	44.4% (4/9)	2.4% (2/85)

a *The number of disruptants verified by PCR analyzed;*

b *The number of transformants*.

## Discussion

In 2008, Maruyama and Kitamoto first described that the multiple gene disruptions with marker recycling were done in the highly efficient gene-targeting background in filamentous fungi (Maruyama and Kitamoto, 2008). They generated a *ligD*(*lig4*)-disruptant for highly efficient gene-disruption frequency in *A. oryzae*., then two proteinase genes(*tppA* and *pepE*) were disrupted continuously at very high frequency (~90%) in Δ*ligD* strain with *pyrG* as the screening marker. After that, *pyrG* has been successfully applied to *Aspergillus terreus* (Huang et al., 2016) and *Aspergillus niger* (Arentshorst et al., 2015) as a selection marker. But, there are no related reports in *Monascus* spp.. In recent years, genes involved in the biosynthesis of citrinin, monacolin K (MK), and pigments, and G protein signaling pathway (Sakai et al., 2009; Li, 2011; Shao et al., 2014; Chen et al., 2017, 2019) have been cloned and analyzed, which made an important step forward in understanding the secondary metabolism in *Monascus* spp.. However, because of the limitation of resistance selection marker genes, multiple-gene editing cannot be performed in the same strain. In this study, based on the issues that there are limited antibiotic screening marker genes available for *Monascus* gene modification and the low GRF (He et al., 2014), *mrpyrG* and *mrlig4* genes from *M. ruber* M7 were knocked out in sequence, leading that a so-called markerlessly and highly efficient gene modification system was successfully constructed without any antibiotic screening marker gene, in which GRF is about 18 times higher than that of *M. ruber* M7. And single or multiple gene(s) related with MPs of *M. ruber* M7 was (were) deleted by this gene modification system.

Although the colonial and microscopic morphologies, biomasses (Figures 3I,III) and CIT production (Figure 4IV) of auxotrophic strains (Δ*mrpyrG* and Δ*pyrG*+*lig4*) and U prototrophic strain (Δ*pyrG*+*lig4*::*pyrG*) on PDA, PDA+U, and PDA+U+5-FOA plates were consistent with those of *M. ruber* M7, U addition could obviously affected the production of orange and red pigments of the U prototrophy strains and some U auxotrophy strains (Δ*mrpyrG*) (Figures 4I,III). Therefore, when the gene markerless modification system constructed in this study is used to investigate the gene(s) function(s) from *Monascus* spp., especially the function(s) of related gene(s) in the MPs gene cluster, it is necessary to re-introduce *pyrG* into the mutants without *pyrG* to avoid the extra addition of U. And why U addition has an effect on the production of the orange and red pigments needs to further be explored.

When the Δ*pyrG*+*lig4* strain from *M. ruber* M7 was taken as the starting strain, its GRFs for *mrpigG* and *mrpigI* reached 46.7 and 44.4%, respectively, due to *mrlig4* loss, which is much (18 times) higher than those of *M. ruber* M7 used as the starting strain (Table 3). However, the positive effect of the *mrlig4* mutant of *M. ruber* M7 on the GRF did not reach the level observed in some other fungi, which GRFs of almost 100% were obtained (Ishibashi et al., 2006; Bugeja et al., 2012). There are also studies that showed inactivation of the *ku70* or *ku80* genes involved in the NHEJ DNA repair pathway can greatly increase GRFs of filamentous fungi (Zhang et al., 2011; He et al., 2013). Therefore, in the future the GRFs may be further improved if the relative genes, such as *mrku70* or/and *mrku80*, with NHEJ pathway, is/are knocked out. Moreover, the characteristics of the multi-gene mutants Δ*pyrG*+*lig4*+*pigG*+*pigH*+*pigI* and Δ*pyrG*+*lig4*+*pigG*+*pigH*+*pigI*::*pyrG* should be investigated, too.

In this study, we construct a markerlessly and highly efficient gene modification system successfully to knock out endogenous *mrpyrG* and *mrlig4* gene without introducing any antibiotic screening marker gene, in which GRF is about 18 times higher than that of *M. ruber* M7. Besides, we have successfully applied this system to multiple-gene knock out in *Monascus* spp.. However, in our study, we also found that the U addition can make an effect on the yield of MPs, and the mechanism is not clear, which requires our further study.

## Data availability statement

The original contributions presented in the study are included in the article/supplementary materials, further inquiries can be directed to the corresponding author/s.

## Author contributions

NX and LL conceived, designed, and did research. NX wrote the manuscript, too. FC revised the manuscript. All authors read and approved the manuscript.

## Funding

This work was supported by the National Natural Science Foundation of China (Nos. 31730068 and 31330059).

## Conflict of interest

The authors declare that the research was conducted in the absence of any commercial or financial relationships that could be construed as a potential conflict of interest.

## Publisher's note

All claims expressed in this article are solely those of the authors and do not necessarily represent those of their affiliated organizations, or those of the publisher, the editors and the reviewers. Any product that may be evaluated in this article, or claim that may be made by its manufacturer, is not guaranteed or endorsed by the publisher.
